# Altered juvenile hormone dynamics mediate earlier foraging in *Nosema*-infected honey bees

**DOI:** 10.1242/bio.062830

**Published:** 2026-09-17

**Authors:** Huarong Lin, Joseph Sullivan, Thomas C. Webster, Zachary Y. Huang

**Affiliations:** ^1^ Department of Entomology, Michigan State University, East Lansing, MI 48824, USA; ^2^ Department of Entomology, University of Illinois at Urbana-Champaign, Urbana, IL 61801, USA; ^3^ College of Agriculture, Health and Natural Resources, Kentucky State University, Frankfort, KY 40601, USA

**Keywords:** *Apis mellifera*, *Nosema apis*, Juvenile hormone, Corpora allata, Behavioral maturation, Precocious foraging, Survival analysis

## Abstract

Parasites can profoundly alter host behavior, yet the physiological mechanisms underlying these changes are often unclear. In honey bees (*Apis mellifera*), microsporidian infection has long been associated with precocious foraging, a life-history shift that can destabilize colony function. Here, we identify the endocrine mechanism linking infection to behavioral maturation. Using *Nosema apis*, we show that infected workers initiate foraging significantly earlier than controls across four colonies (hazard ratio=2.69, *P*<0.001). This behavioral acceleration was accompanied by a ∼1.7-fold increase in circulating juvenile hormone (JH) titers, an ∼83% increase in JH biosynthesis by the corpora allata (CA), and increased *in vivo* JH metabolism, causing elevated JH turnover during infection. Crucially, surgical removal of the CA eliminated the significant effect of infection on foraging, supporting the hypothesis that activation of this gland contributes to the behavioral effects of infection. Together, these results reveal that microsporidian infection accelerates honey bee behavioral maturation by increasing JH turnover through activation of the CA.

## Teaser

**Summary:** We show that *Nosema apis* does not produce juvenile hormone directly but rather causes early foraging via increased hormone synthesis, resulting in higher hormone titers, despite increased metabolism.

## INTRODUCTION

Parasites are well recognized as agents capable of reshaping host behavior, sometimes in ways that enhance parasite transmission or persistence (Moore, 2002; Adamo, 2013). Despite numerous examples of parasite-induced behavioral changes, the physiological mechanisms underlying these effects remain poorly understood. In social insects, colony function depends on tightly regulated division of labor (Wilson, 1971). Consequently, parasite-induced changes in individual behavior have the potential to scale up to affect colony-level organization and performance.

In honey bees (*Apis mellifera*), worker behavior follows a well-characterized developmental trajectory in which individuals perform in-nest tasks early in life and transition to foraging at older ages, a process termed age polyethism (Seeley, 1982) or behavioral development (Robinson, 1987). This shift is highly plastic and responds to both colony needs and individual physiological state (Huang and Robinson, 1996). Accelerated maturation can increase short-term foraging effort but often comes at the cost of reduced lifespan and diminished colony resilience (Perry et al., 2015).

A central regulator of this transition is juvenile hormone (JH). Experimental elevation of JH induces precocious foraging (Robinson, 1987), while JH titers reliably distinguish nurses from foragers even when age is decoupled from behavior (Robinson et al., 1989). Rates of JH biosynthesis by the corpora allata (CA) closely track circulating hormone levels (Huang et al., 1991), and removal of the CA delays the onset of foraging (Sullivan et al., 2000). Together, these findings establish the CA–JH axis as a key endocrine pathway controlling division of labor in honey bees. JH titers are determined not only by biosynthesis in the CA but also by hormone degradation and clearance; thus, multiple physiological processes can potentially regulate behavioral maturation. Microsporidian parasites of the genus *Nosema* infect adult honey bees and have long been associated with altered physiology (Wang and Moeller, 1971) and behavior (Wang and Moeller, 1970). Early studies reported that *Nosema apis* infection accelerates the onset of foraging (Hassanein, 1953; Wang and Moeller, 1970), suggesting that infection perturbs life-history scheduling. More recently, the globally prevalent *Nosema ceranae* has been linked to shortened lifespan (Milbrath et al., 2015), metabolic disruption (Mayack and Naug, 2009; 2010), and earlier foraging (Goblirsch et al., 2013; MacInnis et al., 2025), along with changes in endocrine markers such as vitellogenin and JH. However, it remains unclear whether infection alters JH dynamics through biosynthesis stimulation in the CA, modification of hormone metabolism or clearance, or via broader physiological pathways acting upstream of, or independently from, the CA.

Distinguishing among these alternative mechanisms is important because increases in circulating JH titers could arise through fundamentally different physiological processes. Infection could stimulate JH biosynthesis by activating the CA, reduce hormone degradation or clearance, alter nutritional or metabolic state sufficiently to accelerate maturation indirectly, or influence upstream regulatory pathways controlling endocrine activity. If parasites accelerate behavioral maturation primarily through activation of the CA–JH axis, they would be acting directly on one of the central endocrine systems regulating division of labor (Robinson, 1987; Guidugli et al., 2005; Elekonich and Roberts, 2005). Alternatively, endocrine changes could represent downstream consequences of infection-induced physiological stress rather than the primary mechanism driving behavioral maturation. Distinguishing between these possibilities requires linking behavioral changes to endocrine mechanisms and determining whether the CA are required for infection-induced behavioral maturation.

Here, we combine behavioral, hormonal, and experimental manipulations to test the hypothesis that microsporidian infection accelerates honey bee behavioral maturation by altering JH dynamics through the CA. Using *N. apis*, we (1) quantify age at first foraging across multiple colonies, (2) measure hemolymph JH titers together with rates of JH biosynthesis and metabolism, and (3) use allatectomy to determine whether the CA are required for infection-induced behavioral changes. Together, these experiments identify a mechanistic pathway linking parasite infection to endocrine regulation of division of labor.

## RESULTS

### Experiment 1: *N. apis* infection accelerates foraging onset

Across four colonies, infected workers initiated foraging earlier than controls (Fig. 1). In a Cox proportional hazards model stratified by colony, infection significantly increased the hazard of first foraging (hazard ratio=2.69, 95% CI 1.99–3.65, *P*<0.001; *n*=532 workers, 196 first-foraging events).

**Fig. 1.**
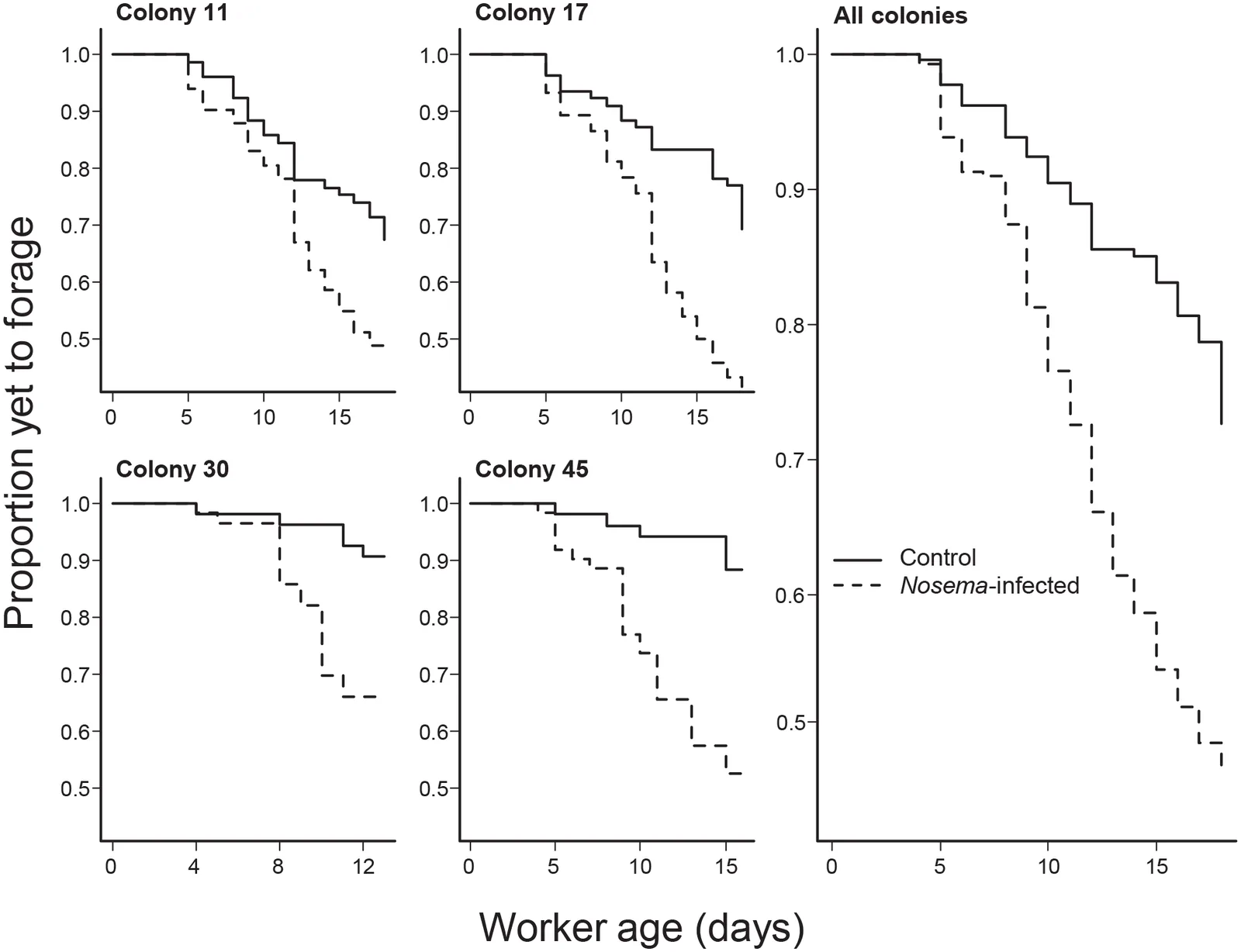
***Nosema* infection accelerated the onset of foraging.** Kaplan–Meier curves show the proportion of workers not yet initiating foraging as a function of age. Left panel: colony-specific curves (four colonies); each colony showed significant differences between the two groups of bees (*P*<0.05). Right panel: pooled across colonies (control versus infected); statistical inference used a Cox model stratified by colony (hazard ratio=2.69, *P*<0.001). *N*=532 workers from four bee colonies, with 196 first-foraging events.

### Experiment 2: *N. apis* infection alters JH dynamics

In both colonies, infected workers exhibited higher hemolymph JH titers than controls (Fig. 2A). A linear mixed model on log-transformed JH titers with colony as a random effect showed that infection significantly increased circulating JH levels [treatment effect=0.53±0.17 standard error of the mean (SE); *t*=3.10; *P*=0.003]. This corresponds to a ∼1.7-fold increase in circulating JH in infected workers [exp(0.534)=1.71].

**Fig. 2.**
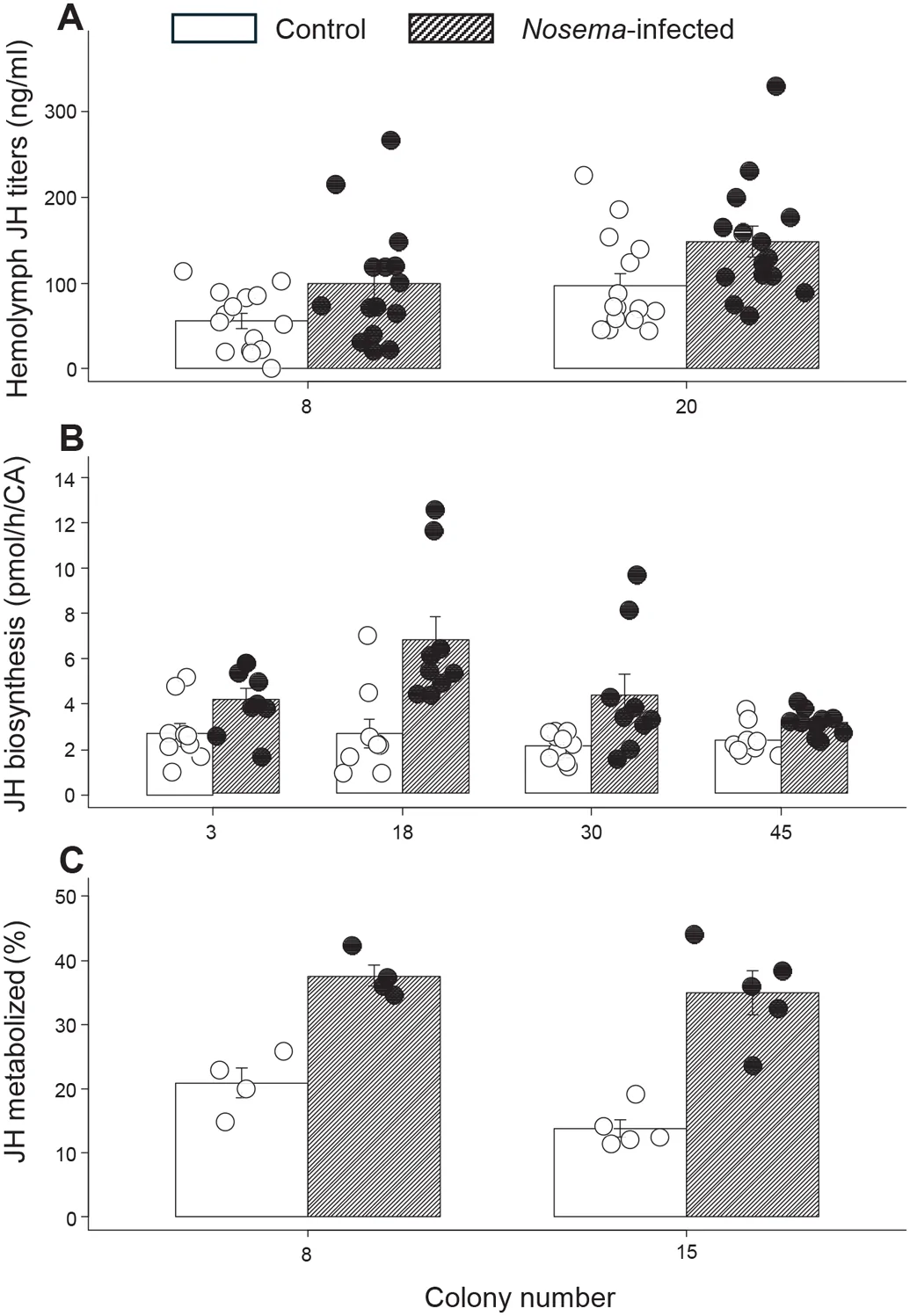
***N. apis* infection altered JH dynamics.** (A–C) *Nosema* infection-elevated hemolymph JH titers (A), JH biosynthesis by the CA (B) and *in vivo* JH metabolism (C). Each point represents one individual worker (biological replicate), and bars show mean±s.e. Data were collected from two independent source colonies (A,C) or four independent source colonies (B). JH titers are expressed as ng ml^−1^ (pg µl^−1^) hemolymph, biosynthesis rates as pmol JH h^−1^ per pair of CA, and metabolism as the percentage of recovered radiolabel present in the metabolized fraction. JH titers and biosynthesis rates were analyzed using linear mixed models on log-transformed values with colony as a random effect, whereas JH metabolism was analyzed using a linear model with colony as a fixed effect. Infection increased circulating JH 1.7-fold and JH biosynthesis by 83% (both *P*<0.01).

Consistent with elevated circulating JH, infection also increased CA JH biosynthesis rates measured *in vitro* across four colonies (Fig. 2B). A linear mixed model on log-transformed biosynthesis rates with colony as a random effect detected a strong effect of infection (treatment effect=0.61±0.11 SE; *t*=5.75; *P*<0.001), corresponding to an approximately 83% increase in JH biosynthesis in infected workers [exp(0.605)=1.83].

In the two colonies examined, *N. apis*-infected workers metabolized a greater proportion of JH than controls (Fig. 2C). A linear model including treatment and colony as fixed effects showed a significant effect of infection (F_1,16_=70.28, *P*<0.001) and a smaller effect of colony (F_1,16_=4.66, *P*=0.046). The model-estimated treatment effect was an increase of 19.2 percentage points (±2.3 SE; *t*=8.38), approximately doubling the proportion of JH metabolized (from ∼17% in controls to ∼36% in infected workers). Thin-layer chromatography (TLC) showed a reduction in the parent JH fraction accompanied by an increase in the putative JH diol metabolite fraction (Fig. S1). Thus, infection increased JH metabolism despite elevated circulating JH titers.

### Experiment 3: allatectomy attenuates infection effects on foraging

Recovery following allatectomy (on day 7) was modestly reduced in *N. apis*-infected bees (76.8%) compared to control bees (85.8%) [binomial generalized linear model (GLM): treatment estimate=−0.622±0.299 SE, *z*=−2.08, *P*=0.037, odds ratio=0.54, 95% CI 0.30–0.96], whereas colony had no significant effect (*P*=0.43). In the recovered workers, *N. apis* infection did not significantly affect the probability of foraging (binomial GLM: treatment estimate=0.366±0.267 SE, *z*=1.37, *P*=0.170, odds ratio=1.44, 95% CI 0.86–2.43, Fig. 3). The overall colony effect was also not significant (likelihood ratio χ^2^=4.08, *P*=0.130).

**Fig. 3.**
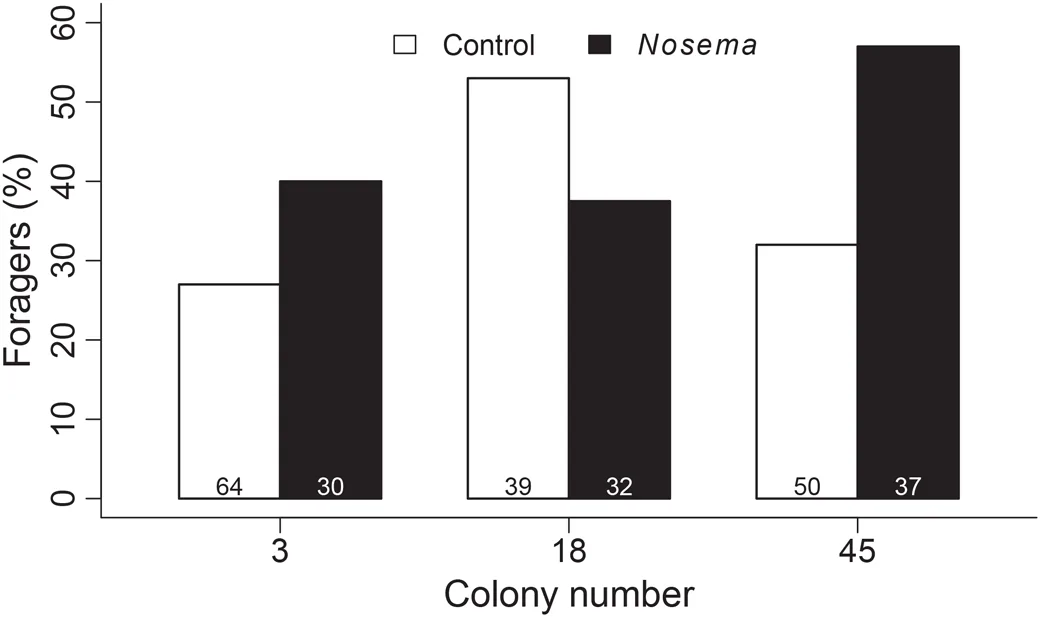
**Allatectomy attenuated *N. apis* infection effects on foraging.** Bars show the percentage of recovered allatectomized workers that initiated foraging by the end of the observation period in three independent source colonies. Numbers within the bars indicate the number of recovered workers available for behavioral analysis in each treatment group. Infection effects were analyzed using a binomial generalized linear model with colony as a fixed effect; the infection effect was not significant (*P*=0.170).

## DISCUSSION

We identify a mechanistic pathway by which a parasite accelerates behavioral maturation in a social insect. Infection with *N. apis* increased JH biosynthesis by the CA, elevated circulating JH titers, and advanced the onset of foraging, despite increased JH metabolism. Removal of the CA eliminated the significant acceleration of foraging associated with infection, consistent with the hypothesis that activation of the CA contributes to infection-induced behavioral maturation. Together, these findings identify the CA–JH axis as a key physiological pathway linking microsporidian infection with division of labor in honey bees.

Our results also clarify how *Nosema* modifies host endocrine physiology. Previous studies suggested that microsporidian infection may elevate JH titers but did not establish the endocrine mechanisms responsible for these changes. For example, Ares et al. (2012) reported consistently higher JH III concentrations in *Nosema*-infected bees using liquid chromatography–tandem mass spectrometry, but statistical analyses were not presented, while Goblirsch et al. (2013) observed similar trends that did not reach statistical significance. Our results extend these observations by providing statistically significant evidence that *N. apis* infection elevates circulating JH titers while simultaneously increasing CA's JH biosynthesis and *in vivo* JH metabolism.

Previous work showed that allatectomized workers retain the capacity to respond to methoprene with accelerated behavioral development (Sullivan et al., 2000). Thus, removal of the CA does not eliminate the downstream behavioral response to JH. If *N. apis* produced JH or a JH-like compound directly, infection would be expected to accelerate foraging even after allatectomy. Instead, infection after allatectomy no longer produced a significant acceleration, supporting the hypothesis that *N. apis* acts by stimulating the host CA rather than by supplying a parasite-derived hormonal signal.

Targeting the CA–JH axis has important implications for colony organization. JH is a central regulator of division of labor (Robinson, 1987; Robinson et al., 1989), and altering its dynamics effectively shifts the timing of behavioral maturation. By accelerating the nurse-to-forager transition, infection may increase short-term foraging effort while reducing worker lifespan and, ultimately, colony resilience (Perry et al., 2015). Thus, endocrine changes in individual workers have the potential to scale up to colony-level consequences.

Although allatectomy eliminated the significant behavioral acceleration associated with infection, this experiment should be interpreted cautiously because removal of the CA may itself influence worker physiology and behavior (Sullivan et al., 2000). Nevertheless, the absence of a consistent infection effect following allatectomy, together with the observed increases in JH biosynthesis and circulating JH titers in intact bees, is consistent with the hypothesis that the CA–JH axis contributes to infection-induced behavioral maturation. Although Experiment 3 lacked a concurrent intact or sham-operated group, all three single-drone-inseminated source colonies used in the allatectomy experiment were also represented in experiments with intact CA. Colonies 3, 18 and 45 were among the four colonies included in Experiment 2B (Fig. 2B), in which infection significantly increased JH biosynthesis across colonies. In addition, workers from Colony 45 exhibited accelerated foraging in Experiment 1 (Fig. 1). Thus, the genetic sources used in the allatectomy experiment were demonstrably responsive to infection under intact conditions. Most importantly, Sullivan et al. (2000) showed that allatectomized bees, although delayed in foraging, could be restored to normal foraging age by methoprene (a JH analog) application. Thus, these bees retained competence to respond to JH and should still show earlier foraging if *N. apis* produced JH or JH-like compounds. Nevertheless, because these measurements were done in different host colonies (during behavioral observation), they do not fully substitute for concurrent intact controls.

Interestingly, although infection modestly reduced recovery following allatectomy, it did not significantly accelerate foraging among surviving workers. This suggests that pathological effects of infection can persist even when the endocrine pathway regulating behavioral maturation is disrupted.

Accelerated behavioral maturation may also have important implications for parasite transmission. By advancing the onset of foraging, *Nosema* infection increases the proportion of workers performing activities outside the colony, where opportunities for pathogen spread are greater. In addition, *Nosema*-infected bees exhibit reduced homing ability (Kralj and Fuchs, 2010; Wolf et al., 2014), perhaps partly due to reduced flight ability (Liu et al., 2026), and infected bees that drift between colonies perform more frequent, although shorter, drifting events (Bordier et al., 2017). Together with the poorer foraging performance of precocious foragers (Perry et al., 2015), these behavioral changes could increase opportunities for movement among neighboring colonies, an important route for pathogen transmission. Recent work further demonstrated that virus-infected bees are more readily accepted by guards of foreign colonies, facilitating inter-colony transmission (Geffre et al., 2020). Although we did not examine drifting or guard acceptance directly, we have independently observed that honey bees infected with *N. ceranae* are more readily accepted by guards of foreign colonies (Li et al., 2026 preprint). Combined with reduced homing ability (Kralj and Fuchs, 2010; Wolf et al., 2014) and altered drifting behavior (Bordier et al., 2017), these observations suggest that parasite-induced acceleration of behavioral maturation could facilitate horizontal transmission among colonies.

Although this study examined *N. apis*, the endocrine mechanism identified here is likely to be relevant to other microsporidian infections, particularly *N. ceranae*, which has become the globally prevalent honey bee microsporidian (Higes et al., 2007). Both species have been associated with precocious foraging and reduced lifespan (Goblirsch et al., 2013; Milbrath et al., 2015; MacInnis et al., 2025), raising the possibility that modulation of the CA–JH axis represents a common mechanism by which microsporidia alter host life history. Determining whether *N. ceranae* similarly increases JH biosynthesis and turnover will be an important direction for future research.

More broadly, these findings contribute to a growing understanding of how parasites alter host behavior by acting on key physiological regulators. In social insects, where individual behavior is integrated into colony-level organization, altering endocrine pathways that regulate division of labor provides an effective means of changing collective behavior. Our results demonstrate that microsporidian infection reshapes honey bee behavioral maturation through the host endocrine system, identifying the CA–JH axis as a central physiological target of parasite-induced behavioral change.

## MATERIALS AND METHODS

### Bees and infections

Worker bees were obtained from multiple source colonies and individually tagged at emergence with numbered plastic tags (Chr. Graze, Germany). Unless otherwise stated, newly emerged workers (<24 h after emergence, designated as 1 day old bees) were assigned to control or infected treatments on the day of emergence. Control bees received 2 µl of 50% sucrose, whereas infected bees received 45,000 *N. apis* spores suspended in 2 µl of 50% sucrose. Bees were isolated individually for 30 min after feeding to minimize cross-contamination (inoculated) and standardize handling (for control). Experiments were conducted in 2001–2002 at Michigan State University (Lansing, MI, USA). Original stock of *N. apis* was obtained from infected field bees near Kentucky State University around 1990 and has since been amplified in the laboratory by feeding it to caged bees. New spores were obtained by inoculating newly emerged bees and harvesting midguts on day 8 each week. This strain of laboratory culture was confirmed to be *N. apis* by PCR analysis in 2006.

### Experiment 1: age at first foraging

Workers from four source colonies, each headed by a single-drone-inseminated queen, were used to assess age at first foraging. Newly emerged bees were tagged and introduced into nucleus colonies (∼5000 workers with a queen). From day 5 onward, colonies were monitored daily, and the age at first foraging was recorded for each individual. A foraging trip was defined as an excursion lasting >5 min (Winston and Katz, 1982).

### Experiment 2A: JH titers

Bees from two colonies were used to measure hemolymph JH titers. Tagged bees from Colony 8 were introduced into a single-cohort nucleus colony containing a queen, one empty frame, and one frame with honey and pollen. To promote foraging, ∼1200 newly emerged bees (1–3 days younger than tagged bees) were added. Tagged bees from Colony 20 were introduced into an observation hive (∼3500 workers, one brood frame, and one food frame) that had been established 1 month prior to the experiment.

At 12 days of age, tagged bees were sampled for JH analysis. Within each treatment, foragers and non-foragers were sampled in proportion to their occurrence in the population. Bees were immobilized on ice, and hemolymph was collected with glass capillaries (Huang and Robinson, 1996), transferred to 500 µl acetonitrile (high-performance liquid chromatography grade), and stored at −20°C until radioimmunoassay. JH radioimmunoassay was performed as described by Jassim et al. (2000).

### Experiment 2B: JH biosynthesis by the CA

Newly emerged workers (120–150 per group) from four single-drone-inseminated colonies were marked on the thorax, fed either spores or syrup, and introduced into four colonies. At 8–11 days of age, bees were collected from within colonies, were immobilized on ice, and had their CA dissected.

JH biosynthesis rates were measured *in vitro* using a radiochemical assay following Huang et al. (1991). Briefly, the CA–corpora cardiaca complex was incubated in a medium containing radiolabeled methionine; synthesized JH was extracted with organic solvent and quantified by liquid scintillation counting.

### Experiment 2C: *in vivo* JH metabolism

Seventeen-day-old infected and control workers from two colonies were used to assess *in vivo* JH metabolism. Each bee received a 1 µl injection of vegetable oil containing 13,500 dpm of radiolabeled JH III (NET586, PerkinElmer). Bees were incubated at 35°C for 1.5 h before homogenization in 0.02 M phosphate buffer (pH 7.0). Radiolabeled JH and products were extracted and separated by TLC using hexane:ethyl acetate:glacial acetic acid (66:33:1, Venkatesh et al., 1988). Because the tritium label was located at C-10 of JH III, it was retained in the major metabolic products, including JH acid and JH diol, allowing metabolism to be quantified by following the redistribution of radioactivity after TLC separation. Following TLC separation, the lane was scraped into 11 equal sections, and the radioactivity in each section was determined by liquid scintillation counting. Overall recovery of radioactivity exceeded 80%.

### Experiment 3: allatectomy and foraging behavior

Bees that emerged recently (0–6 h) were allatectomized under a stereomicroscope following Sullivan et al. (2000). Following surgery, bees were allowed to recover for 8–12 h at 32°C before *Nosema* infection. Bees were infected at the same dose and method as in Experiment 1.

Groups of treated and control bees (40–70 per group) from the same source colony were introduced into host colonies (∼10,000 workers). Colony entrances were screened for 6 days to prevent premature foraging while flight muscles developed. Observations for foraging of the two groups of bees started when focal bees were 7–23 days old. The experiment was replicated across three colonies.

### Statistical analyses

For Experiment 1, colony was included as a stratification factor in the Cox proportional hazards model. For Experiments 2A and 2B, colony was included as a random effect in linear mixed models. For Experiment 2C, colony was included as a fixed effect because only two colonies were available. For Experiment 3, colony was included as a fixed effect in the generalized linear model because only three colonies were available. All analyses were conducted in R (version 4.5.2, R Core Team, 2024); scripts used for analyses and figures are provided at https://doi.org/10.5281/zenodo.21855021.

### Use of artificial intelligence

ChatGPT (OpenAI, GPT-5.5) was used for assistance in checking R statistical models and code and for English grammar and spelling. All statistical analyses, code, scientific interpretation, and manuscript content were verified and approved by the authors.

## Supplementary Material



10.1242/biolopen.062830_sup1 Supplementary information
