# Supplementary material for: Altered juvenile hormone dynamics mediate earlier foraging in Nosema-infected honey bees
Source: Biol Open. 2026 Sep 17;15(9):bio062830. doi: 10.1242/bio.062830 (PMC13617973; doi:10.1242/bio.062830)
Supplement: Supplementary file 1 [file biolopen-15-062830-s1.pdf]

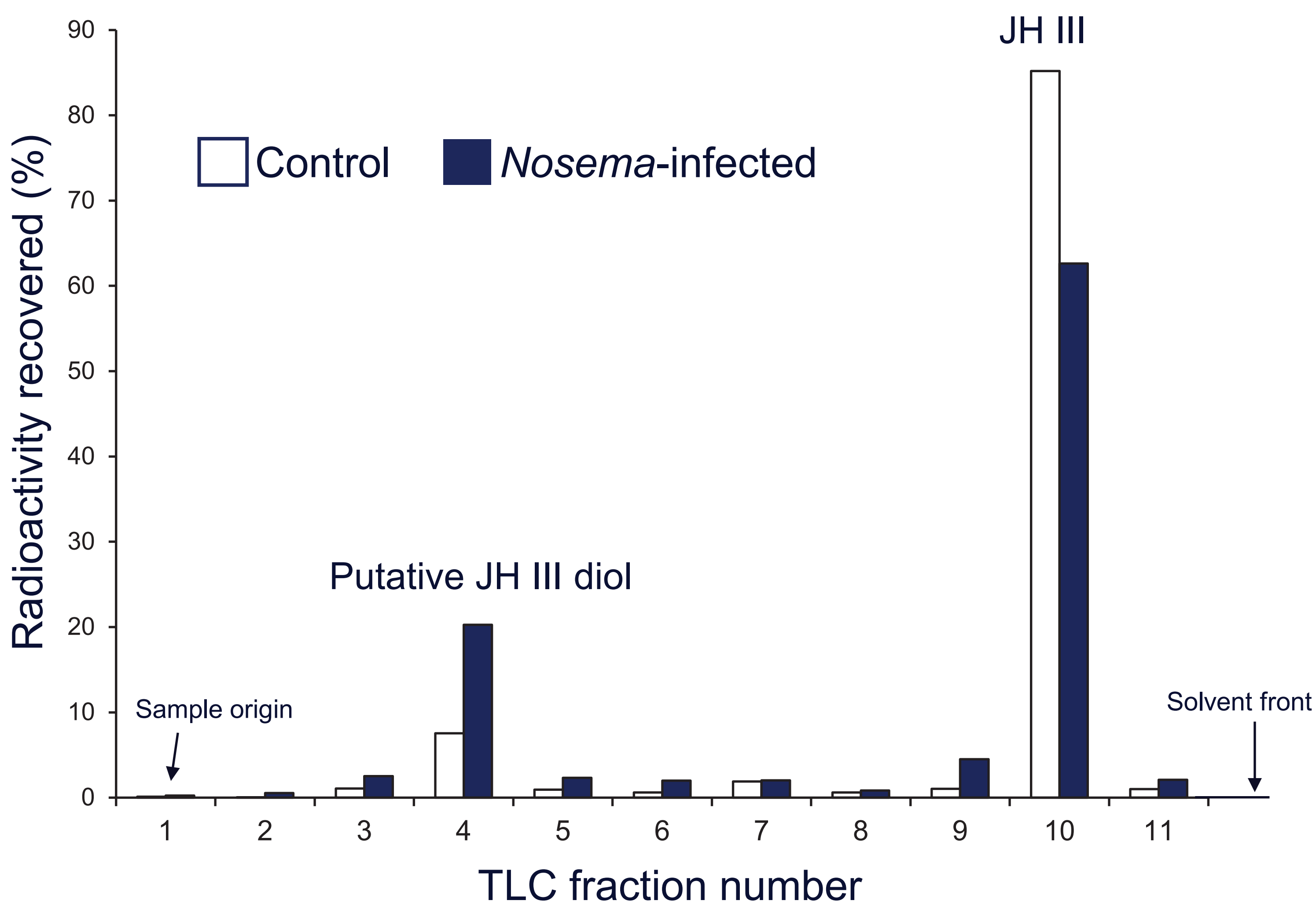

Fig. S1. Representative thin-layer chromatogram of recovered radioactivity following injection of radiolabeled JH III into bees and incubation for 1.5 h. Fraction 10 corresponded to unmetabolized JH III, as verified by chromatography of the radiolabeled JH III standard on the same TLC plate. Fraction 4 corresponded to the putative JH III diol peak, based on chromatographic mobility using identical TLC solvent system described by Venkatesh et al. (1988). Nosema-infected bees exhibited reduced radioactivity in the parent JH III peak and increased radioactivity in the putative JH III diol peak, indicating enhanced JH metabolism.a
