# Supplementary material for: Altered juvenile hormone dynamics mediate earlier foraging in Nosema-infected honey bees
Source: Biol Open. 2026 Sep 17;15(9):bio062830. doi: 10.1242/bio.062830 (PMC13617973; doi:10.1242/bio.062830)
Supplement: Supplementary file 2 [file biolopen-15-062830_review_history.pdf]

# Altered juvenile hormone dynamics mediate earlier foraging in Nosema-infected honey bees

Ron Lin, Joseph Sullivan, Thomas Webster and Zachary yong Huang
10.1242/bio.062830

**Editor**: Kendra J. Greenlee

**Review timeline**
Original submission: 23 July 2026
Editorial decision: 30 July 2026
First revision received: 10 August 2026
Accepted: 15 August 2026

---

**Original submission**

First decision letter

MS ID#: bio.062830

MS Title: Altered juvenile hormone dynamics mediate earlier foraging in Nosema-infected honey bees

Authors: Zachary yong Huang, Ron Lin, Joseph Sullivan and Thomas Webster

I have now reached a decision on the above manuscript.

The reviewer reports are shown at the bottom of this email.

**As you will see, the reviewers raised a number of substantial criticisms that prevent me from accepting the paper at this stage.**

They suggest, however, that a revised version might prove acceptable, if you can address their concerns. If you think that you can deal satisfactorily with the criticisms on revision, I would be pleased to see a revised manuscript. In particular, both reviewers noted concerns about the data in Fig. 3. The suggestion of reviewer 1 for a positive control is valid. However, I realize that these experiments were conducted several years ago, so it would not be possible to repeat the experiment. Is it possible to present the data in the same way as in Fig. 1? I agree with reviewer 2 who recommends including additional data from the workers in Fig. 3, such as survival, recovery rate, or behavioral changes. In the figure legend for Fig. 3, the title states that *Nosema* infection attenuated the earlier foraging. However, in two of the three colonies, the percentage of foragers was higher in infected bees, whereas only one colony showed a decrease in the percentage of infected foragers. In addition, that colony had a higher than expected percentage of foragers from the control allatectomized bees. Although the statistical test results found no effect of infection, it seems that was likely due to having only three colonies with very different outcomes. I would echo the reviewer suggestion about including discussion about the limitations of the data presented in Fig. 3

Additionally, in the methods section, the descriptions of Experiment 1 and 2A do not mention how or when bees were infected.

© 2026. Published by The Company of Biologists under the terms of the Creative Commons Attribution License (https://creativecommons.org/licenses/by/4.0/).

Might this paper explain some of the increase in JH metabolism? Takatsuka, J., Nakai, M., & Shinoda, T. (2017). A virus carries a gene encoding juvenile hormone acid methyltransferase, a key regulatory enzyme in insect metamorphosis. Scientific Reports, 7(1), 13522. doi:https://doi-org.ezproxy.lib.ndsu.nodak.edu/10.1038/s41598-017-14059-8

At this stage, we also ask you to ensure your manuscript complies with our formatting guidelines. Provided you are able to fully address the referees' comments, we are positive about publication of your paper (we accept over 95% of revision submissions) and therefore hope you won't mind any extra work involved in reformatting your manuscript at this point.

Please upload both a 'clean' version of your Word file, along with a highlighted version clearly showing where you have made changes in the revised manuscript. Please avoid using 'Track changes' in Word files as these are lost in PDF conversion.

I should be grateful if you would also provide a point-by-point response detailing how you have dealt with the points raised by the reviewers in the 'Response to Reviewers' box. Please attend to all of the reviewers' comments. If you do not agree with any of their criticisms or suggestions please explain clearly why this is so.

Reviewer 1

Comments for the author

In this manuscript, Lin et al investigated how infection of honey bees with microsporidia Nosema apis causes earlier foraging compared to uninfected controls. The authors found that infected bees had increased levels of circulating juvenile hormone, an increase in juvenile hormone synthesis by the corpora allata, and elevated JH metabolism. Additionally, when the authors surgically remove corpora allata, the accelerating effect microsporidial infection on foraging was not present, suggesting that corpora allata is required for the effect of infection on behaviour. The authors test a clearly stated biological hypothesis, and overall, the experimental design is well suited to test that hypothesis. The manuscript is well-written and conclusions are mostly supported by the data. I have one major and few minor suggestions to clarify and strengthen the manuscript.
Major point
Figure 3. Here, the authors examined the effect of infection on foraging in bees with removed corpora allata and claim that in these animals infection had no significant effect. However, this experiment lacks a positive control (demonstration that infection triggered foraging behaviour in unmanipulated (with corpora allata) animals). What if under given experimental conditions (age, feeding, recovery, etc) infection does not induce foraging behaviour irrespective of corpora allata manipulation? Given that in this experiment the authors used different protocol then in Figure 1 and show data in a different way, we can't simply assume that the same effect on foraging will be present under experiment 3. Positive control is necessary to show that bees with intact gland treated under the same conditions as the ones with removed gland do actually exhibit behavioural changes after infection.
Minor points
1. Supplementary figure 1. Can the authors explain how they know which fraction contains what?
2. Figure 2 legend. The authors should explain the units shown on the graphs (ng/ml, …)
3. Revising the references and citing more recent publications is encouraged.
4. At least two other studies (https://pmc.ncbi.nlm.nih.gov/articles/PMC3590174/ https://www.sciencedirect.com/science/article/pii/S1570023212002887) have shown that microsporidial infections trigger an increase in juvenile hormone in bees. Those should be appropriately discussed, as those supporting the findings of a current manuscript. Also, the claim in 235-236 is not appropriate as this has been done already.

Reviewer 2

Comments for the author

This manuscript examines whether Nosema apis infection accelerates behavioral maturation in worker honey bees through altered juvenile hormone dynamics and activity of the corpora allata. The topic is important because infection-induced shifts in worker behavior could affect both

© 2026. Published by The Company of Biologists under the terms of the Creative Commons Attribution License (https://creativecommons.org/licenses/by/4.0/).

individual physiology and colony-level organization. The integration of behavioral observations, hormone measurements, biosynthesis and metabolism assays, and surgical manipulation is a clear strength; overall, the experiments are generally well designed and the manuscript is well written.

However, I think there are a couple of important issues in the manuscript that should be addressed. First, the Introduction should more fully develop plausible alternative mechanisms by which infection could alter maturation, including effects on JH degradation or clearance, metabolic and nutritional pathways, and mechanisms acting upstream of or independently from the corpora allata. These are currently briefly introduced in the Discussion, but I think expanded discussion of alternative mechanistic hypotheses in the Introduction would help readers and clarify the value of the manuscript (and justify the specific experiments). Second, the allatectomy experiment also requires more cautious interpretation and expanded discussion, because CA removal or surgery may broadly affect survival, recovery, metabolism, flight, and foraging capacity, potentially obscuring treatment differences. Finally, the statistical methods should more explicitly describe how colony-level variation was modeled, report complete test statistics, and address whether differential survival, recovery, or detection could have influenced the results.

I have included specific comments on these and other aspects of the manuscript below.

Specific comments
L74-80: The discussion of alternative hypotheses here needs to be expanded. In particular, alternative hypotheses for how infection could alter development (now generaly described as 'infection-induced stress or broader physiological disruption') need to be discussed in more detail, and in particular considering whether there are plausible mechanisms that might not involve the CA (e.g. altered degradation of JH rather than production, other drivers of maturation, etc).

L90: Was there any differential survival between these treatments? I.e. did infected bees have higher mortality, or were they less likely to be detected in subsequent experiments?

L100: Here and elsewhere - 'Experimental 1' should be either 'Experiment 1' or potentially 'Experimental Design 1' if that was the intended meaning

L137: Here, was recovery differential between infection treatments?

L143-146: What was the survival and recovery rate for this manipulation? And it is not clear from this description when the data on foraging were collected (and specifically what age were the bees when foraging was observed? This is important for interpreting the foraging effects in Fig 3)

L147: Did statistical models used here account for colony level variation, e.g. including colony as a random effect in models?

L161: I know this is the correct statistical terminology, but I would suggest rewording to clarify the direction of the results for its biological meaning here

L184 - 187: where there other effects of the allatectomy? E.g. did workers recover, otherwise behave normally after CA removal, etc? Given that the main results here is a a lack of effect on foraging (and it appears that overall foraging rate in these experiments was low), is there a possibility that the CA removal (or the procedure itself) disrupted foraging or behavior more broadly (wiping out treatment effects as a result)? In particular, previous work from the authors has I believe shown that allatectomy can have generic effects on flight flight, foraging and metabolism:

Sullivan, et al. "Juvenile hormone and division of labor in honey bee colonies: effects of allatectomy on flight behavior and metabolism." Journal of Experimental Biology 206.13 (2003): 2287-2296.

L199-201: I think it would be helpful to include some of the discussion of these alternative pathways in the introduction

L183-187: The inclusion of colony level effects isn't described in the models above, and also full test statistics (instead of just p values) should be reported

© 2026. Published by The Company of Biologists under the terms of the Creative Commons Attribution License (https://creativecommons.org/licenses/by/4.0/).

L219: What is meant by 'poorer' here? Reduced efficiency? Activity levels?

L340: Here and elsewhere: 'Nosema' should be italicized. I might also suggest including the species (Nosema apis) throughout, or abbreviated to N. apis

Reviewer's Responses to Questions

**Experimental quality**
Are experiments performed using appropriate methods that will answer the question (or test the hypothesis or support the observations) posed by the authors? Is the right tool used for the job?
*If 'No', please indicate reasons in Comments for Author box below.*
Reviewer #1:

- No

Reviewer #2:

- Yes

---

Were the data analyzed using appropriate statistical tests?
*If 'No', please indicate reasons in Comments for Author box below.*
Reviewer #1:

- Yes

Reviewer #2:

- Yes

---

**Reproducibility**
Were experiments performed using adequate number of biological replicates?
*If 'No', please indicate reasons in Comments for Author box below.*
Reviewer #1:

- Yes

Reviewer #2:

- Yes

---

Is there sufficient raw data to assess the rigor of the analysis?
*If 'No', please indicate reasons in Comments for Author box below.*
Reviewer #1:

- Yes

Reviewer #2:

- Yes

---

Does the methods section provide sufficient detail to permit reproducibility?
*If 'No', please indicate reasons in Comments for Author box below.*
Reviewer #1:

- Yes

Reviewer #2:

- Yes

---

**Completeness**
Are the manuscript's conclusions supported by the data?
*If 'No', please indicate reasons in Comments for Author box below.*

© 2026. Published by The Company of Biologists under the terms of the Creative Commons Attribution License (https://creativecommons.org/licenses/by/4.0/).

Reviewer #1:
- No

Reviewer #2:
- Yes

---

**Scholarship**
Do the authors cite and discuss the merits of data that would argue for and against their conclusion?
*If 'No', please indicate reasons in Comments for Author box below.*
Reviewer #1:
- No

Reviewer #2:
- Yes

---

Does the manuscript title & abstract accurately reflect the contents of the manuscript, without hyperbole?
*If 'No', please indicate reasons in Comments for Author box below.*
Reviewer #1:
- Yes

Reviewer #2:
- Yes

---

**First revision**

Author response to reviewers' comments

**Dear Editor,**

We thank the Editor and both reviewers for their careful and constructive evaluations. We have revised the manuscript to clarify the alternative endocrine mechanisms considered, strengthen reporting of the statistical analyses, acknowledge the limitations of the allatectomy experiment, add relevant recent literature, and moderate our causal interpretation where appropriate. Below, each reviewer comment is reproduced in italics, followed by our response and the location of the corresponding revision.

Line numbers refer to the track-change version (v14) that is submitted. But the numbers might be slightly off since many changes are being made while this response was still being in progress.

**Reviewer 1**
*Figure 3. Here, the authors examined the effect of infection on foraging in bees with removed corpora allata and claim that in these animals infection had no significant effect. However, this experiment lacks a positive control (demonstration that infection triggered foraging behaviour in unmanipulated (with corpora allata) animals). What if under given experimental conditions (age, feeding, recovery, etc) infection does not induce foraging behaviour irrespective of corpora allata manipulation? Given that in this experiment the authors used different protocol then in Figure 1 and show data in a different way, we can't simply assume that the same effect on foraging will be present under experiment 3. Positive control is necessary to show that bees with intact gland treated under the same conditions as the ones with removed gland do actually exhibit behavioural changes after infection.*

© 2026. Published by The Company of Biologists under the terms of the Creative Commons Attribution License (https://creativecommons.org/licenses/by/4.0/).

**Response:** We agree that a concurrent sham-operated group would have strengthened the design by controlling for nonspecific effects of surgery. However, such a control would not by itself establish whether allatectomized workers remain capable of responding to a JH signal. This critical physiological control was demonstrated previously by Sullivan et al. (2000), who showed that methoprene accelerated behavioral development in allatectomized workers. Thus, removal of the corpora allata does not abolish the downstream capacity to respond to JH or to initiate foraging. The allatectomies in the present study were performed by the same investigator (J. Sullivan) using the same established procedure. Consequently, if *Nosema* acted by producing JH or a JH-like compound directly, infection would still be expected to accelerate foraging in CA-removed workers. The absence of a significant acceleration therefore supports the interpretation that infection acts primarily through stimulation of the host CA-JH axis. We nevertheless retain cautious wording because the present experiment did not include a concurrent sham-operated group.

We now state that the allatectomy results are consistent with, and support, involvement of the CA–JH axis rather than conclusively proving that the gland is required. We also clarify an important feature of the design: Colony 45 in Experiment 3 was derived from the same single-drone-inseminated queen and was studied in the same field season as Colony 45 in Experiment 1. In Experiment 1, workers from this same genetic background with intact corpora allata showed a strong infection-associated acceleration of foraging. In addition, all three colonies in Exp 3 were used for Exp 2B, which showed JH biosynthesis rate changes in colonies 3, 18, 30, 45. This demonstrates that the source genotypes were responsive to infection under intact conditions, although it does not fully substitute for a concurrent sham-operated group. Finally, we explicitly discuss the possibility that surgery or CA removal could influence flight, metabolism, recovery, or foraging capacity independently of the infection effect.

**Changes in the manuscript:** The main interpretation is softened in the Discussion (lines 277-283) and the limitation and same-genotype evidence are discussed at lines 284-291. The Abstract was also softened at lines 33–34 by replacing “demonstrating that this gland is required” with “supporting the hypothesis that activation of this gland contributes to the behavioral effects of infection.”

1. *Supplementary Figure S1: explain how the authors know which fraction contains JH and which contains its metabolites.*

**Response:** We thank the reviewer for requesting clarification. We have expanded the Methods and Supplementary Figure S1 legend to explain how metabolite identity was assigned. The position of parent JH III was established by direct chromatography of radiolabeled JH III. The metabolite peak was assigned as the putative JH III diol fraction based on chromatographic mobility using the identical TLC solvent system and methodology reported by Venkatesh et al. (1988). We also clarify that the tritiated H label was located at C-10 of JH III and was retained in the principal metabolic products, ensuring that redistribution of radioactivity accurately reflects JH metabolism.
Our data: see Supplementary Fig. 1S. Our putative JH diol was at 4 and JH at 10.
Venkatesh et al data, using the same solvent system. Their JH diol was at 10 with JH at 30.

Figure provded for the reviewers in confidence has been removed

2. *The Figure 2 legend should explain the units shown on the graphs.*

**Response:** We agree. The revised legend defined the units for each panel: hemolymph JH titer in ng ml−1, CA biosynthesis in pmol h−1 CA−1, and JH metabolized as a percentage of recovered radioactivity. JH titers and rates of biosynthesis followed the regular units Huang and others have published previously.

3. *The references should be revised and more recent publications cited.*

**Response:** We have updated the scholarship in both the Introduction and Discussion. The revision now cites recent and directly relevant work on infection-associated behavioral maturation and physiology, impaired homing, altered drifting, and inter-colony acceptance, including Ares et al. (2012), Wolf et al. (2014), Bordier et al. (2017), Geffre et al. (2020), MacInnis et al. (2025), and our

© 2026. Published by The Company of Biologists under the terms of the Creative Commons Attribution License (https://creativecommons.org/licenses/by/4.0/).

other study on higher guard acceptance of nosema infected bees (Li et al., 2026). These additions place the endocrine findings within the broader behavioral and epidemiological literature.

**Changes in the manuscript:** Recent literature is incorporated at lines 64–76, 204–221, and 243–261.

4. *At least two previous studies showed that microsporidian infection increases JH in bees. These studies should be discussed, and the claim that this had not been done previously should be removed.*

**Response:** We thank the reviewer for bringing these studies to our attention. We have now cited and discussed both Ares et al. (2012) and Goblirsch et al. (2013). We also revised our wording to avoid implying that elevated JH during microsporidian infection had not previously been investigated. We note, however, that Ares et al. reported descriptive JH concentrations without formal statistical analyses, whereas Goblirsch et al. reported trends toward elevated JH titers that did not reach statistical significance. The present study extends these previous observations by providing statistically supported evidence that *N. apis* infection significantly elevates circulating JH titers and demonstrating increased JH biosynthesis by the CA, together with increased JH metabolism.

### Reviewer 2

1. *First, the Introduction should more fully develop plausible alternative mechanisms by which infection could alter maturation, including effects on JH degradation or clearance, metabolic and nutritional pathways, and mechanisms acting upstream of or independently from the corpora allata. These are currently briefly introduced in the Discussion, but I think expanded discussion of alternative mechanistic hypotheses in the Introduction would help readers and clarify the value of the manuscript (and justify the specific experiments).*

**Response:** We agree and substantially expanded the Introduction. It now explains that identical increases in circulating JH could result from increased CA biosynthesis, reduced degradation or clearance, altered nutritional or metabolic state, or upstream pathways that regulate endocrine activity. This framing provides the rationale for separately measuring circulating JH, CA biosynthesis, and in vivo JH metabolism. Changes made in lines 78–93, especially lines 82–93.

In addition we have now revised the discussion extensively.

2. *Second, the allatectomy experiment also requires more cautious interpretation and expanded discussion, because CA removal or surgery may broadly affect survival, recovery, metabolism, flight, and foraging capacity, potentially obscuring treatment differences*

Please see our response to Reviewer one's first question. An important physiological control was already provided by Sullivan et al. (2000) – in that study they showed a delay of CA-removed bees in foraging, but this delay can be reversed by methoprene application, thus proving that honey bees without CA are still capable of flight and foraging. We do agree if we had sham-operated bees also in control and infected groups, it would increase our confidence. However, we did the foraging observation the same way as in Experiment 1. And the same method of analysis also yielded a negative results (please see answer to question 7 below). Lastly, the 3 source colonies providing workers in Exp 3 also were used in Exp 2B, showing these same bees did respond to Nosema infection by showing higher JH biosynthesis. *Most importantly, the Sullivan et al (2000) study already showed that the CA-removed bees, although showed delayed foraging, could be restored to normal-aged foraging by methoprene (a JH analog) application. Thus these bees still have competence to respond to JH and should show earlier foraging if* N. apis *produced JH or chemicals with JH-like effects.*

3. *Finally, the statistical methods should more explicitly describe how colony-level variation was modeled, report complete test statistics, and address whether differential survival, recovery, or detection could have influenced the results.*

© 2026. Published by The Company of Biologists under the terms of the Creative Commons Attribution License (https://creativecommons.org/licenses/by/4.0/).

We now make these explicitly. We revised in the methods: "For Experiment 1, colony was included as a stratification factor in the Cox proportional hazards model. For Experiments 2A and 2B, colony was included as a random effect in linear mixed models. For Experiment 2C, colony was included as a fixed effect because only two colonies were available. For Experiment 3, colony was included as a fixed effect in the generalized linear model because only three colonies were available."

We now report slight differences in recovery for Experiment 2C, but using all recovered bees we detected no earlier foraging. Please see answer to question 6 below.

4. *L74-80: The discussion of alternative hypotheses here needs to be expanded. In particular, alternative hypotheses for how infection could alter development (now generaly described as 'infection-induced stress or broader physiological disruption') need to be discussed in more detail, and in particular considering whether there are plausible mechanisms that might not involve the CA (e.g. altered degradation of JH rather than production, other drivers of maturation, etc).*

   **Response:** We thank the reviewer for this helpful suggestion. We substantially expanded the Introduction to discuss several plausible mechanisms by which microsporidian infection could accelerate behavioral maturation. Specifically, we now explain that elevated circulating JH could arise through increased biosynthesis by the CA, reduced hormone degradation or clearance, alterations in nutritional or metabolic state, or changes in upstream endocrine pathways regulating the CA. These alternative hypotheses provide the rationale for measuring not only circulating JH titers, but also JH biosynthesis and in vivo JH metabolism. The Introduction now explains how the present experiments distinguish among these competing mechanisms rather than assuming a priori that the corpora allata are the primary target of infection.

5. *L90: Was there differential survival between treatments, or were infected bees less likely to be detected subsequently?*

**Response:** Recovery during the first week after introduction did not differ detectably between treatments. The modest reduction in recovery reported in the revised manuscript refers specifically to recovery following allatectomy in Experiment 3.
(please see answer to number 7 below).

6. *L100 "Experimental 1" should be "Experiment 1" or "Experimental Design 1."*

**Response:** We apologize for the typos. They were corrected throughout to "Experiment 1," "Experiment 2," and "Experiment 3."

7. *L137: Here, was recovery differential between infection treatments?*
   *L143-146: What was the survival and recovery rate for this manipulation? And it is not clear from this description when the data on foraging were collected (and specifically what age were the bees when foraging was observed? This is important for interpreting the foraging effects in Fig 3)*

**Response:** We now report recovery rates in the results section. We now add: "Recovery following allatectomy (on day 7) was modestly reduced in *N. apis*-infected bees (76.8%) compared to control bees (85.8%) (binomial GLM: treatment estimate = −0.591 ± 0.297 SE, z = −1.99, P = 0.047), whereas colony had no significant effect (P = 0.43). In the recovered workers, *N. apis* infection did not significantly affect the probability of foraging (binomial GLM: treatment estimate = 0.366 ± 0.267 SE, z = 1.37, P = 0.170; odds ratio = 1.44, 95% CI = 0.86–2.43, Fig. 3). The overall colony effect was also not significant (likelihood-ratio $x^2_2$ = 4.08, P = 0.130)." Thus, *Nosema* infection retained measurable pathological effects following allatectomy. However, despite this reduction in recovery, infection did not significantly accelerate foraging among bees that recovered, supporting the conclusion that CA removal specifically attenuated the behavioral effect of infection.

Our data on foraging was observed from day 7 to day 23, now added into the methods.

© 2026. Published by The Company of Biologists under the terms of the Creative Commons Attribution License (https://creativecommons.org/licenses/by/4.0/).

To further evaluate the reviewer's concern, we also entered the data of ages at first foraging for all recovered allatectomized workers and performed a complementary Cox proportional hazards analysis, analogous to Experiment 1. This analysis likewise detected no significant overall effect of infection (hazard ratio = 1.32, 95% CI = 0.88–1.98, P = 0.176). Furthermore, a treatment × colony interaction model detected no significant interaction (likelihood-ratio test, P = 0.103), indicating that the response to infection did not differ significantly among colonies. Thus, both the original analysis and the survival analysis support the conclusion that infection did not significantly accelerate foraging after allatectomy. Because this complementary analysis was performed in response to the review and does not address the absence of concurrent sham-operated control, we have not incorporated it into the manuscript.

**Response:** We agree. The revised Statistical analyses section now states explicitly that colony was used as a stratification factor in Experiment 1, as a random effect in the mixed models for Experiments 2A and 2B, and as a fixed effect for Experiments 2C and 3 because only two or three colony levels were available. The Results now report estimates, standard errors, test statistics, confidence intervals, degrees of freedom and P-values where appropriate.

8. *Reword the statistical terminology to make the biological direction of the effect clear.*

**Response:** We revised the Results to state directly that infected workers metabolized a greater proportion of JH than controls and that the treatment effect corresponded to an approximately 19.2 percentage-point increase in the proportion metabolized (Lines 210-211). This wording presents the biological direction before the statistical details.

*L184 - 187: where there other effects of the allatectomy? E.g. did workers recover, otherwise behave normally after CA removal, etc? Given that the main results here is a a lack of effect on foraging (and it appears that overall foraging rate in these experiments was low), is there a possibility that the CA removal (or the procedure itself) disrupted foraging or behavior more broadly (wiping out treatment effects as a result)? In particular, previous work from the authors has I believe shown that allatectomy can have generic effects on flight, foraging and metabolism:*

**Response:** We agree that this is an important limitation. We now cite Sullivan et al. (2000) and explicitly state that allatectomy itself can affect worker physiology and behavior. Consequently, we interpret the lack of a consistent infection effect after allatectomy as supportive, but not definitive, evidence for CA–JH involvement. However, these CA-removed bees did perform foraging, just slightly delayed similarly to those reported by Sullivan et al (2000). More importantly, in that same study they showed the delayed foraging can be restored by application of methoprene, a JH analog. We also call for future experiments with sham-operated controls. Changes are in Lines 273 to 287.

9. *Discussion of alternative pathways should also appear in the Introduction.*

**Response:** Done. The expanded Introduction (See lines 66–97) now presents altered biosynthesis, metabolism, clearance, nutritional state, and upstream endocrine regulation before stating the study hypothesis.

10. *What is meant by “poorer” foraging performance?*

**Response:** We revised this discussion to specify the relevant phenotypes rather than using the vague term “poorer.” The revised paragraph refers to reduced homing ability, altered drifting behavior, and reduced foraging performance, with supporting citations to Kralj and Fuchs (2010), Wolf et al. (2014), Bordier et al. (2017), and Perry et al. (2015). These changes were in lines 289-305.

11. *Nosema should be italicized, and the species should be given throughout as Nosema apis or N. apis.*

**Response:** We agree. We have standardized taxonomic usage so that full species names are given at first mention and abbreviated thereafter.

© 2026. Published by The Company of Biologists under the terms of the Creative Commons Attribution License (https://creativecommons.org/licenses/by/4.0/).

Second decision letter

MS ID#: bio.062830R1

MS Title: Altered juvenile hormone dynamics mediate earlier foraging in Nosema-infected honey bees

Authors: Zachary yong Huang, Ron Lin, Joseph Sullivan and Thomas Webster

I appreciate the extensive revisions that you made to your manuscript and the thorough response to reviewers. I am happy to tell you that your manuscript has been accepted for publication in Biology Open, pending our standard publication integrity checks. It was accepted on 15th August 2026.

© 2026. Published by The Company of Biologists under the terms of the Creative Commons Attribution License (https://creativecommons.org/licenses/by/4.0/).
